# NtDREB-1BL1 Enhances Carotenoid Biosynthesis by Regulating Phytoene Synthase in *Nicotiana tabacum*

**DOI:** 10.3390/genes13071134

**Published:** 2022-06-24

**Authors:** Chen Dong, Qingdong Wang, Yubo Wang, Lili Qin, Yongchun Shi, Xiaoran Wang, Ran Wang

**Affiliations:** 1 College of Life Sciences, Henan Agricultural University, Zhengzhou 450002, China; chen.dong@haut.edu.cn (C.D.); shiyongchun@henau.edu.cn (Y.S.); xiaoranwang@henau.edu.cn (X.W.); 2 College of Biological Engineering, Henan University of Technology, Zhengzhou 450001, China; qin19971204@163.com; 3 Henan Key Laboratory of Bioactive Macromolecules, Laboratory of Straw Enzymatic Technology Research, College of Life Science, Zhengzhou University, Zhengzhou 450001, China; qdwang@zzu.edu.cn (Q.W.); 15736778920@163.com (Y.W.)

**Keywords:** antioxidants, carotenoid biosynthesis, DREB, drought stress, phytoene synthase, transcription factor

## Abstract

As one of the most imperative antioxidants in higher plants, carotenoids serve as accessory pigments to harvest light for photosynthesis as well as photoprotectors for plants to adapt to high light stress. Phytoene synthase (PSY) is the entry enzyme and also the major rate-limiting enzyme in the carotenoid pathway. Here, we report a dehydration-responsive element-binding protein (DREB) transcription factor member in *Nicotiana tabacum* K326, NtDREB-1BL1, which regulates carotenoids biosynthesis by binding to the *NtPSY* promoter. The *NtDREB-1BL1* transcript was widely distributed in leaves by Real-time PCR. Confocal image revealed that NtDREB-1BL1 was localized in the nucleus. The chromatin immunoprecipitation (ChIP) with the qPCR technique indicated that NtDREB-1BL1 could anchor the promoter region of *NtPSY*. Overexpression (*NtDREB-1BL1 OE*) and RNA interference (*NtDREB-1BL1 RNAi*) of *NtDREB-1BL1* were performed to evaluate its biological function in *N. tabacum*. Both carotenoid and chlorophyll contents increased in transgenic plants of *NtDREB-1BL1 OE* compared with wild-type (WT) plants, with the augment of the genes involved in carotenoid biosynthesis. In contrast, the contents of carotenoid and chlorophyll significantly decreased in transgenic plants of *NtDREB-1BL1 RNAi* compared to WT, along with the decline in the expression of genes related to carotenoid biosynthesis. Moreover, transgenic plants of *NtDREB-1BL1 OE* exhibited enhanced tolerance under drought stress, with the weakened tolerance of drought stress in transgenic plants of *NtDREB-1BL1 RNAi*. In conclusion, our results illustrated the new role of transcription factor NtDREB-1BL1 in improving carotenoid biosynthesis through regulating *NtPSY* expression.

## 1. Introduction

Carotenoids are essential nutrients and important health-beneficial compounds for humans. As a subgroup of isoprenoid compounds with diverse structures, carotenoids are widely distributed in plants, algae, photosynthetic bacteria, and fungi [1,2,3,4,5]. Carotenoids are well-known antioxidants in higher plants, which not only quench reactive oxygen species (ROS) but also serve to sacrifice themselves under conditions of oxidative stress [2,5]. Moreover, carotenoids play imperative roles in plant growth, development, photosynthesis, and the defense against various stresses. Therefore, regulating carotenoid biosynthesis is of great significance to the production and quality of crops [6,7,8,9,10]. The mevalonate pathway (MVA) and the 2-C-methyl- D-erythritol-4-phosphate (MEP) pathway are found in plants for generating terpenoids. The MVA pathway has evolved to produce steroids and sesquiterpene in the cytoplasm, while the MEP pathway exists in plastids to synthesize carotenoids, monoterpenes, diterpenoids, and other compounds [11].

Phytoene synthase (PSY) catalyzes the condensation of two geranylgeranyl diphosphate (GGPP) to synthesize phytoene. Catalyzing the first committed step in the carotenoid biosynthesis pathway, PSY is a major rate-limiting enzyme of carotenogenesis [2]. *PSY* expression can be regulated by phytochrome [12]. The *SlPSY* gene in tomato (*Solanum lycopercicum*) exhibits functional differentiation and tissue-specific expression. Compared with the *SlPSY1* gene playing function in tomato fruit, the *SlPSY2* gene is mainly involved in photosynthesis, photoprotection, and the synthesis of abscisic acid precursors in the leaves [13]. During the ripening of tomato fruit, phytochrome regulates the accumulation of carotenoids in the fruits through the expression of the *SlPSY* gene [14]. Moreover, *OsPSY1* and *OsPSY2* genes in rice are specifically induced by light signals [15].

Several transcription factors have been found to regulate *PSY* expression. The phytochrome-interacting factor 1 (PIF1) is able to repress *PSY* expression by binding to the *PSY* promoter. However, PIF1 is degraded after the interaction with photoactivated phytochromes under the high light condition, which rapidly increases *AtPSY* expression along with the carotenoid accumulation [16]. In addition, the lycopene content in tomato is regulated by the photoreceptors of transcription factors HY5 and PIF, showing a certain connection between light signal and expression of genes related to carotenoid biosynthesis [17,18,19]. Carotenoid content in various plants, including tomato, wheat, and peach, significantly increases when *PSY* is overexpressed [20,21,22]. Considering the essential role in regulating *PSY* expression, the exploration of new transcription factors is a crucial way to reveal genetic mechanisms for regulating the carotenoid pathway.

As a unique transcription factor of plants, the DREB family actively participates in the response process to various stresses, such as drought, low temperature, and salt [23]. The ethylene response factor (RAP2.2) belongs to the ethylene response element-binding protein (ERF) transcription factor family and regulates carotenoid content in higher plants by anchoring the *PSY* promoter [24]. DREB factors or CRT element binding factors (CBFs) are members of the AP2/ERF family, which comprise a large number of stress-responsive regulatory genes [25,26,27]. The overexpression of *DREB* in *Arabidopsis* effectively enhances the resistance of transgenic plants to low temperature, drought, and high salt, exhibiting physiological changes related to cold adaptation, such as dwarfing of transgenic plants [28]. *AtDREB1B* transgenic tomato also show dwarfing phenomenon, but exogenous gibberellin (GA) prevents the appearance of the dwarfing phenotype [29]. Compared with the wild type, the genetically modified tomatoes treated with GA have a relatively weak resistance to drought and low temperature [28].

To date, few *DREB* genes have been characterized from the tobacco genome, and whether DREB genes are involved in carotenoid biosynthesis has not been reported so far. Based on our screening results of the initial yeast one-hybrid experiment, it was initially discovered that NtDREB-1BL1 was predicted to interact with the *PSY* gene promoter in *N. tabacum*. Exploring the regulation of carotenoid biosynthesis by *NtDREB-1BL1* has great theoretical significance in improving the stress resistance and quality of crops, providing a theoretical foundation for the genetic regulation of carotenoid content.

## 2. Materials and Methods

### 2.1. Plant Materials and Growth Conditions

The seeds of *N. tabacum* K326 and *N. benthamiana* collected from Henan Agricultural University, were cultured in a greenhouse at 26 °C with a photo-cycle of 16 h light/8 h dark, and the relative humidity was (60 ± 2)% as growth conditions previous described [30,31]. The growth conditions of tobacco cultured in Murashige–Skoog (MS) medium were 16 h/8 h of the light-dark cycle, and the temperature was (23 ± 2) °C. The light intensity was 50 μmol m^−2^ s^−1^. Seeds of *N. tabacum* and *N. benthamiana* were retained in the laboratory. The leaves, stems, roots, and flowers of *N. tabacum* were collected and stored at −80 °C. For drought stress, 40-day tobaccos with the same physiological condition were selected and grown in normal conditions without watering for 8 days. Tobacco plants were treated with 300 mM NaCl solution for 6 h [32]. After stress treatment, the leaves of tobaccos were collected and stored in a refrigerator at −80 °C for later use.

### 2.2. Identification and Analysis of NtDREB

The yeast one-hybrid experiment (Y1H) was performed as the reported method with some modification [33]. Briefly, the promoter of the *NtPSY* gene (GenBank ID: JF461341) as a bait was cloned into baited vector pHIS2 (Clontech, Mountain View, CA, USA). The recombinant vector was transformed into the yeast Y1H Gold strain (Clontech, Mountain View, CA, USA). Moreover, a total of 1 g of tobacco leaves was crushed, and total RNA was extracted using Plant RNA Purification Reagent (Sangon, Shanghai, China). The first-strand cDNA was synthesized by RNA Reverse Transcription Kit (Thermo Fisher, Waltham, MA, USA), and the amplification was conducted using the Advantage R2 PCR Kit (Clontech, Mountain View, CA, USA). The purified cDNA was mixed with the pGADT7-Rec2 prey vector and transformed into the bait yeast strain containing the promoter of the *NtPSY* gene. Then, the candidate transcription factors were screened by inoculating the library solution. After the reporter gene detection, DNA sequencing and BLAST comparison analysis of positive clones were applied, and the proteins interacting with the *NtPSY* gene were determined. Yeast one-hybrid experiments initially found that the transcription factor gene interacting with the key region of the *NtPSY* gene promoter was NtDREB-1BL1 (GenBank ID: XM_016632493.1).

The homologous proteins of different species were identified from the TIGR rice database (http://rice.plant biology.msu.edu (accessed on 10 June 2021)) and *Arabidopsis* TAIR (http://arabidopsis.org (accessed on 10 June 2021)) database. The homologous protein sequences of DREB-1BL1 in *N. tomentosiformis*, *N. sylvestris* and *N. benthamiana* were searched in the tobacco genome database (using BLASTP, e < 0.001). The conservative domain online prediction software SMART version 9 (http://smart.embl-heidelberg.de/ (accessed on 15 June 2021)) was used to analyze the sequence and confirm that all candidate proteins had typical conserved domains [34]. The GSDS version 2.0 online software (http://gsds.cbi.pku.edu.cn/ (accessed on 20 June 2021)) was performed to visualize gene structure and annotated features. Online software HMMER (https://www.ebi.ac.uk/Tools/hmmer/ (accessed on 25 July 2021)) was used to study the transmembrane domain of candidate proteins [35]. The molecular weight, isoelectric point, and other physical properties of candidate proteins were predicted by Expasy online software (http://web.expasy.org/protparam/ (accessed on 12 August 2021)) [36]. The DREB-1BL1 candidate protein sequences of different species were compared by the Clustal W program. Mega 7.0 was performed (neighbor-joining algorithm, 1000 bootstrap replicates) for sequence alignment and inference of evolutionary tree [37]. The promoter region of *NtPSY* was analyzed by Plant Care software. (https://www.plantcare.co.uk/ (accessed on 17 August 2021)) [38].

### 2.3. Subcellular Localization of NtDREB-1BL1

The *NtDREB-1BL1* gene was amplified by PCR from the cDNA of *N. tabacum* leaves with the primers (Table S1). The overexpression vector of Sp1300-NtDREB-1BL1-GFP was constructed by *Xba*I and *Kpn*I restriction enzymes, which was constructed to express NtDREB-1BL1 with a GFP-tag at the C terminus under the control of the cauliflower mosaic virus 35S promoter (Table S1). The recombinant vectors were transferred into *Agrobacterium* (GV3101) by electroporation and cultured at 30 °C for 2 days. The positive *Agrobacterium* was scraped from the solid dish with the inoculation loop and cultured in 10 mL YEB medium. *Agrobacterium* cells were suspended with 10 mM MgCl_2_ (including 120 μM acetosyringone, AS) and adjusted OD600 to 0.6. *N. benthamiana* plants with about five leaves were infected with Agrobacterium for the transient expression of NtDREB-1BL1-GFP protein. Fluorescence observation was performed by a FluoView FV 1000 laser scanning confocal microscopy system (Olympus, Tokyo, Japan) as described previously [31].

### 2.4. Expression Pattern of NtDREB-1BL1 and Genes Involved in Carotenoid Biosynthesis

Total RNA was isolated from plant tissues using the Plant Total RNA Isolation Kit (Sangon, Shanghai, China), and the first-strand cDNA was synthesized by RNA Reverse Transcription Kit (Thermo Fisher, USA). The expression levels of genes were detected via quantitative real-time PCR (qPCR) using 26s rRNA as the reference gene. The reaction system and conditions were consulted with the manufacturer’s protocol [30]. Three biological replicates were conducted for each sample. The relative expression levels of genes were calculated using the 2^-∆∆CT^ method. The gene-specific primers used for qPCR are listed in Table S1.

### 2.5. Vector Construction and Tobacco Transformation

The vector of Sp1300-NtDREB-1BL1-GFP described above was used for overexpression of *NtDREB-1BL1*. The 200–300 bp fragment of the *NtDREB-1BL1* gene was inserted into the silencing vector pHellsgate 2 by BP recombination reaction. The tobacco genetic transformation process was performed as described previously [31]. Homozygous transgenic (T2) plants of *NtDREB-1BL1 OE* were screened and confirmed by seed germination on the corresponding hygromycin-containing medium in combination with PCR analyses. Moreover, homozygous transgenic (T2) plants of *NtDREB-1BL1 RNAi* were confirmed by seed germination on the corresponding kanamycin-containing medium in combination with PCR analyses.

### 2.6. Chromatin Immunoprecipitation (ChIP) Assay

An EpiQuik Chromatin Immunoprecipitation kit (Epigentek, Farmingdale, NY, USA) was used to determine whether NtDREB-1BL1 could bind to the key segments of the *NtPSY* gene promoter according to reported protocol with some modification [39]. Briefly, two-week-old tobacco seedlings cultured on MS medium were collected and immediately fixed in 1% formaldehyde solution under vacuum infiltration for 10 min. The chromatin was sheared by sonication to break the DNA into 200–1000 bp fragments. The NtDREB-1BL1-GFP fusion protein was pulled down in the strip wells with an Anti-GFP antibody (Abcam, Waltham, MA, USA). DNA fragments were eluted from the fusion protein and examined by the qRT-PCR analyses. The enrichment of the genomic fragment in two *tubulin* *beta chain* genes (NtTUBB, XP_016456097.1, and NP_001312648.1) was used as the negative control. The primers used for fragment amplification are listed in Table S1.

### 2.7. Extraction and Determination of Chlorophyll and Carotenoid

The chlorophyll and carotenoids determinations were performed from transgenic tobacco as described previously [40]. Briefly, 50 mg of leaves were ground into fine powder, and mixed with 80% acetone. The mixture of leaves was protected from light and shocked at 4 °C to bleach completely. The supernatant was harvested by centrifugation at 9000 rpm for 2 min at 4 °C, and the absorbance values at the wavelengths of A663, A647, and A470 were measured by the enzyme marker using 80% acetone as control. Chlorophyll and carotenoid contents were calculated as follows: Chlorophyll a = 12.25 × A663 − 2.79 × A647; Chlorophyll b = 21.50 × A647 − 5.10 × A663; Total chlorophyll = 7.15 × A663 + 18.71 × A647; Carotenoids = (1000 × A470 − 1.82 × Chl a − 85.02 × Chl b) /198.

### 2.8. Reactive Oxygen Species (ROS) Detection

The staining methods used for the DAB detection of H_2_O_2_ and the NBT detection of O_2_^−^ were performed as described previously [30]. The contents of O_2_^−^ and H_2_O_2_ in leaves were analyzed according to the color variations of the leaves. Leaves of transgenic tobaccos from the same position were taken and separately stained with DAB or NBT for 24 h in the dark at 28 °C. The leaves were washed with distilled water to remove DAB or NBT, and repeatedly boiled in 80% ethanol until they were completely decolorized. The leaves were laid on a colorless solid medium for taking photographs. Moreover, ROS in transgenic tobacco was quantified by a colorimetric method using O_2_^−^ and H_2_O_2_ content assay kits (Sangon, China).

### 2.9. Statistical Analysis

Statistical analysis was performed as our previous study [31]. A completely randomized block design with at least three biological replicates was applied for each experiment. The data were represented by the mean ± standard deviation from three biological samples, and further analyzed using the SPSS statistical package (version 8.0). The student’s t test was used for statistical analysis. * and ** indicated that compared with the control, the difference was statistically significant, *p* < 0.05 and *p* < 0.01, respectively.

## 3. Results

### 3.1. Identification and Characterization of NtDREB-1BL1

The *NtDREB-1BL1* gene was identified from *N. tabacum* (XM_016632493.1). The molecular weight and isoelectric point of the protein were shown in Table S2. Homologous genes of the *NtDREB-1BL1* gene were identified in the *N. sylvestris* and *N. tomentosiformis*, which were named as NtomDREB1 and NsyDREB1. The similarity of these three genes was 97.35%, and the similarity of the coding DNA sequences was 96.92%. The *NtDREB-1BL1* gene was 99.54% similar to *NsyDREB1*. NtDREB-1BL1 was rich in hydrophilic amino acid residues, without transmembrane domain. The size of the protein was about 24 kDa. The protein instability coefficient was greater than 40, which indicated that transmembrane domain was necessary for protein stability. The cis-acting element bound by the DNA binding region was DRE/CRT (dehydration responsive element/C-repeat element), and the core sequence was ACCGAC.

### 3.2. Phylogenetic Analysis, Exon-Intron Architecture and Conserved Domains of NtDREB -1BL1

**Figure 1:**
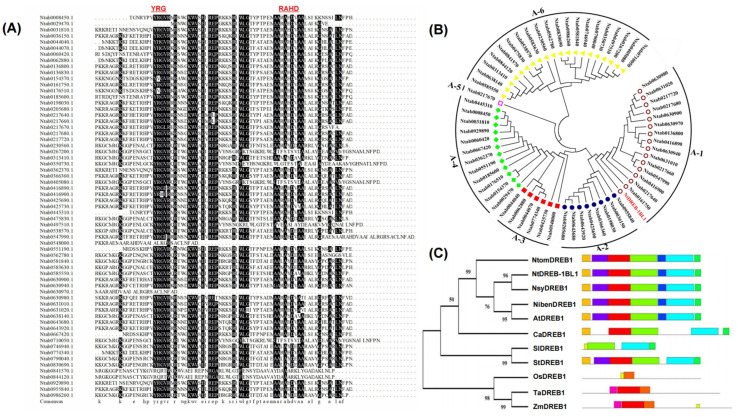
Sequence alignment and phylogenetic analysis of NtDREB family in *N. tabacum*. (**A**) AP2 domain analysis of NtDREB family. (**B**) Phylogenetic analysis of NtDREB family. (**C**) The distribution of conserved motifs in DREB. The protein sequences were from *Nicotiana tomentosiformis* (NtomDREB1); *Nicotiana sylvestris* (NsyDREB1); *Nicotiana tabacum* (NtDREB-1BL1); *Nicotiana benthamiana* (NibenDREB1); *Solanum lycopersicum* (SlDREB1, XP_019068359.1); *Capsicum annuum* (CaDREB1, PHT68900.1); *Arabidopsis thaliana* (AtDREB1, AT4G25490); *Oryza sativa* (OsDREB1, NP_001359120.1); *Zea mays* (ZmDREB1, NP_001105876.2); *Triticum aestivum* (TaDREB1, AAX13289.1); *Solanum tuberosum* (StDREB1, NP_001274894.1). Different color blocks represented various conserved motifs.

DREB belonged to the AP2/EREBP family and contained only one AP2 domain. Based on the combined analysis of gene annotation and conserved domains, NtDREB without the complete AP2 domain was eliminated. A total of 63 genes with the complete AP2 domains (conserved YRG and RAHD regions) were detected (Figure 1A). Phylogenetic analysis indicated that the members of the NtDREB family were divided into six subgroups (Figure 1B). NtDREB-1BL1 was grouped in the A-1 class, containing 16 members. The exon-intron architecture was analyzed by GSDS 2.0 software, indicating that most members of the *NtDREB* family had no introns.

The homologous proteins of NtDREB-1BL1 in tobacco, *Arabidopsis*, wheat, rice, tomato, and potato were analyzed by the maximum likelihood method. NtomDREB1, NsyDREB1, NtDREB-1BL, NibenDREB1, and DREB in *Arabidopsis thaliana* (AtDREB) were closer in the genetic relationship, while DREB in *Capsicum annuum* (CaDREB), *Solanum lycopersicum* (SlDREB) and *Solanum tuberosum* (StDREB), belonging to *Solanaceae,* were closer in the genetic relationship, indicating that DREB were relatively conserved during evolution. The motifs of NtDREB1-1BL1, NtomDREB1, NibenDREB1, NsyDREB1, and AtDREB1 were conserved, all of which were relatively similar to CaDREB. The DREB in *Triticum aestivum* (TaDREB) and *Zea mays* (ZmDREB), which belong to *Poaceae*, exhibited similar motifs (Figure 1C).

### 3.3. Expression Pattern and Subcellular Localization of NtDREB-1BL1

**Figure 2:**
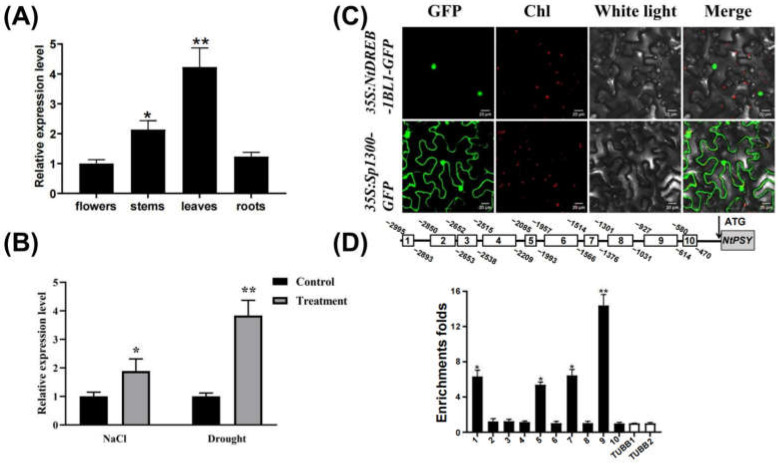
Expression pattern, subcellular localization, and ChIP-qPCR of NtDREB-1BL1. (**A**) The relative pattern of *NtDREB-1BL1* in various tissues. (**B**) Relative expression of *NtDREB-1BL1* in response to drought and NaCl stresses. Relative expression was evaluated by qPCR using 26s rRNA as a reference gene. Asterisks represent statistically significant differences by Student’s *t*-test (**, *p* < 0.01; *, *p* < 0.05). (**C**) Subcellular location of NtDREB-1BL1 proteins fused to GFP (green color) in tobacco. Chl, chlorophyll autofluorescence (red color). (**D**) The enrichment of binding fragments was examined by ChIP-qPCR. The black line represented the 3 kb sequence upstream of the ATG of the *NtPSY* gene. The white boxes represented the DNA fragments amplified in the ChIP assay. ChIP enrichment test of the promoter regions bound by NtDREB-1BL1 was examined by ChIP-qPCR. Asterisks represent statistically significant differences between WT and transgenic plants determined by Student’s *t*-test (**, *p* < 0.01; *, *p* < 0.05).

Real-time qPCR was used to evaluate the expression level of *NtDREB-1BL1* in tissues of leaves, stem, root and flower in *N. tabacum*. The transcript level was the highest in the leaves, with the lowest in the flowers (Figure 2A). Additionally, both NaCl and drought stress induced the up-regulation of *NtDREB-1BL1* in leaves of *N. tabacum* (Figure 2B). Predicted localization of NtDREB-1BL1 by the WoLF PSORT system suggested that NtDREB-1BL1 was most probably localized in the nucleus. Additionally, the transient protein expression system of Agrobacterium-infected tobacco leaves was used to illustrate NtDREB-1BL1 localization. The control tobacco leaves was inoculated by *Agrobacterium* carrying Sup1300-GFP empty vector, whose green fluorescence was mainly distributed in the cytoplasm and nucleus. While the green fluorescence signal of NtDREB-1BL1-GFP fusion protein was only visible in the nucleus (Figure 2C), which was in accordance with the expression pattern of the transcription factor and its possible regulatory role.

### 3.4. ChIP-qPCR Analysis

The Plant Care software was used to analyze the promoter region of the *NtPSY* gene in *N. tabacum*, which was divided into 10 segments, and the distribution of cis-acting elements (Table S3) was analyzed. The specific qPCR primers were designed to identify the association between transcription factors and its target cis-acting elements. The DNA fragments obtained in the ChIP experiment were used as substrates to perform qPCR experiment. As shown in Figure 2D, four fragments containing the binding site of NtDREB-1BL1 in the promoter regions of *NtPSY* genes were significantly enriched after immunoprecipitation. Among these four fragments, the relative expression level of the ninth segment was significantly higher than that of other segments (Figure 2D). The promoters distributed in ninth segment included CAAT-box, TATA-box and MBS, of which CAAT-box was the common cis-acting element in promoter and enhancer regions, and MBS was an important element involved in drought induction (Table S3).

### 3.5. Characterization of NtDREB-1BL1 OE and RNAi Transgenic Tobacco

**Figure 3:**
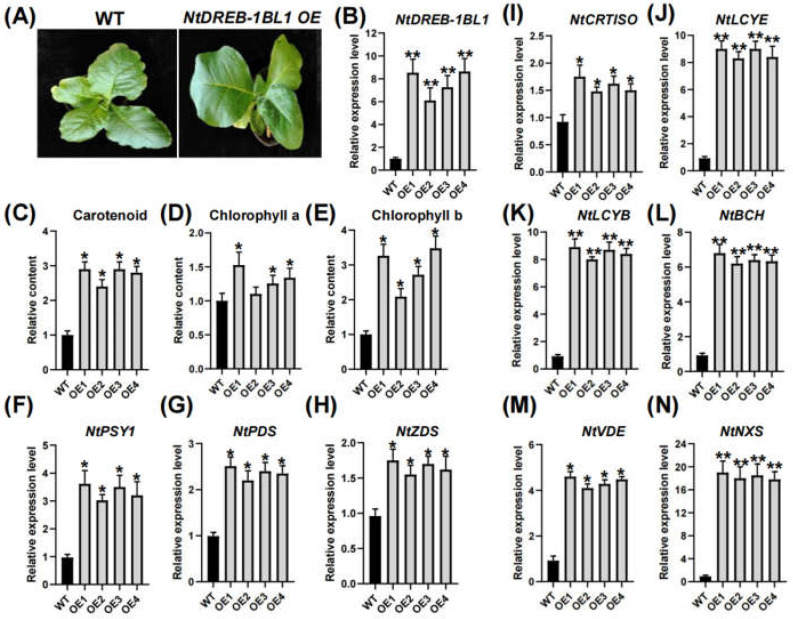
Overexpression of *NtDREB-1BL1* improved carotenoid contents and expression of genes related with carotenoid biosynthesis. (**A**) Phenotype of *NtDREB-1BL1 OE* transgenic tobacco. (**B**) Relative expression of *NtDREB-1BL1* transcript in OE transgenic tobacco. (**C**–**E**) The relative contents of carotenoid, chlorophyll a and chlorophyll b. (**F**–**N**) Relative expression of genes involved in carotenoid biosynthesis including *NtPSY1*, *NtPDS*, *NtZDS*, *NtCRTISO*, *NtLCYE*, *NtLCYB*, *NtBCH*, *NtVDE*, and *NtNXS*. Asterisks represent statistically significant differences between WT and transgenic plants determined by Student’s *t*-test (**, *p* < 0.01; *, *p* < 0.05).

**Figure 4:**
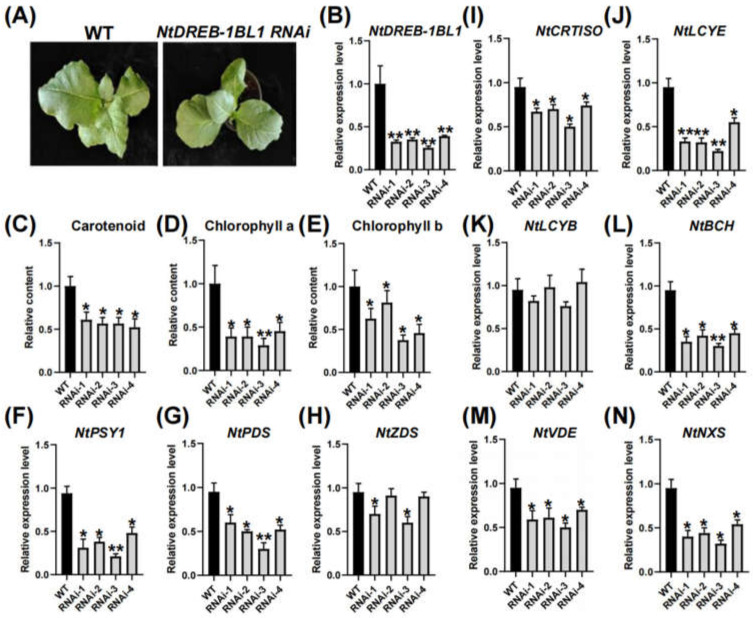
Inhibition of *NtDREB-1BL1* decreased carotenoid contents and expression of genes related to carotenoid biosynthesis. (**A**) Phenotype of *NtDREB-1BL1 RNA*i transgenic tobacco. (**B**) Relative expression of *NtDREB-1BL1* transcript in RNAi transgenic tobacco. (**C**–**E**) Relative contents of carotenoid, chlorophyll a, and chlorophyll b. (**F**–**N**) Relative expression of genes involved in carotenoid biosynthesis, including *NtPSY1*, *NtPDS*, *NtZDS*, *NtCRTISO*, *NtLCYE*, *NtLCYB*, *NtBCH*, *NtVDE*, and *NtNXS*. Asterisks represent statistically significant differences between WT and transgenic plants determined by Student’s *t*-test (**, *p* < 0.01; *, *p* < 0.05).

The *NtDREB-1BL1 OE* transgenic T1 line were obtained (Figure 3A), and four transgenic T1 lines were selected for further analysis. *NtDREB-1BL1* transcripts were significantly increased in *NtDREB-1BL1 OE* transgenic lines (Figure 3B). Pigment analysis indicated that the contents of carotenoids, chlorophyll a and chlorophyll b were increased in *NtDREB-1BL1 OE* transgenic lines (Figure 3C–E). The overexpression of the *NtDREB-1BL1* gene promoted the expression of *NtPSY1*, phytoene dehydrogenase *(NtPDS*), *zeta-carotene desaturase (NtZDS*), *carotenoid isomerase* (*NtCRTISO*), *lycopene beta cyclase* (*NtLCYB)*, *Lycopene epsilon cyclase* (*NtLCYE*), *beta-carotene hydroxylase* (*NtBCH*), *violaxanthin de-epoxidase* (*NtVDE*), and *neoxanthin synthase* (*NtNXS*) in the carotenoid biosynthesis pathway (Figure 3F–N). The *NtDREB-1BL1 RNAi* transgenic T1 line was also investigated (Figure 4A), with the *NtDREB-1BL1* transcript decreased by more than 50% (Figure 4B). The contents of carotenoids, chlorophyll a and chlorophyll b were relatively declined in *NtDREB-1BL1 RNAi* transgenic lines (Figure 4C–E). Moreover, the transcripts of *NtPSY1*, *NtPDS*, *NtZDS*, *NtCRTISO*, *NtLCYE*, *NtBCH*, *NtVDE*, and *NtNXS* in the carotenoid synthesis pathway were relatively decreased, except for *NtLCYB* (Figure 4F–N).

### 3.6. NtDREB-1BL1 Function in Defense Against Drought Stress

**Figure 5:**
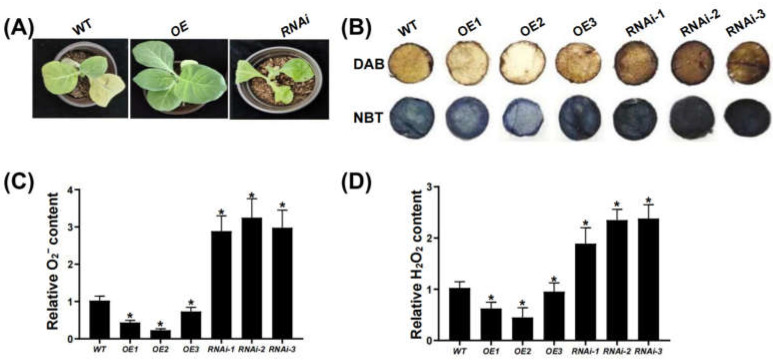
NtDREB-1BL1 has a role in defence against drought stress. (**A**) Phenotype of *NtDREB-1BL1 OE* and *RNAi* transgenic tobacco after drought treatment for 8 days. (**B**) DAB and NBT staining of *NtDREB-1BL1 OE* and *RNAi* transgenic tobacco after drought treatment. (**C**,**D**) The detailed O_2_^−^ and H_2_O_2_ contents in these materials were relatively quantified by the colorimetric method. Asterisks represent statistically significant differences between WT and transgenic plants determined by Student’s *t*-test (*, *p* < 0.05).

The WT, *NtDREB-1BL1 OE* and *RNAi* transgenic tobacco lines with consistent growth were selected for drought treatment. The leaves of control and *NtDREB-1BL1 RNAi* tobacco seedlings were withered, whereas the *NtDREB-1BL1 OE* transgenic seedlings still exhibited healthy condition (Figure 5A). An analysis of the color of the leaves showed that a more dark brown color appeared in *NtDREB-1BL1 RNAi* transgenic tobacco after DAB staining compared with the control (Figure 5B). However, the color of *NtDREB-1BL1 OE* transgenic tobacco leaves was lighter than control tobacco. A similar phenomenon was also detected after NBT staining. Darker blue staining was detected in *NtDREB-1BL1 RNAi* transgenic tobacco compared with control, while the blue was lighter in *NtDREB-1BL1 OE* transgenic leaves. Drought treatment promoted the accumulation of ROS in WT, *NtDREB-1BL1 RNAi* and *OE* transgenic tobacco. However, the increase in O_2_^−^ and H_2_O_2_ was lower in *NtDREB-1BL1 OE* transgenic lines than WT, which was more in *RNAi* transgenic lines than WT (Figure 5C,D). The lower levels of ROS caused by NtDREB-1BL1 indicated that NtDREB-1BL1 conferred enhanced protection from oxidative damage and a better ROS scavenging ability in tobacco.

## 4. Discussion

DREB transcription factors have been successfully transformed into model plants such as *A. thaliana* [41], *O. sativa* [42], *Z. mays* [43,44], and *T. aestivum* [45], improving the tolerance of plants to low temperature, drought, and high salt. The DREB family plays important roles in the response to abiotic stress. However, knowledge of *NtDREB* genes is limited. In this study, the *NtDREB-1BL1* gene was isolated from *N. tabacum*. A detailed study of the classification, phylogenetic evolution, gene structure, and expression pattern of these *NtDREB-1BL1* in response to drought stress was carried out (Figure 1). As an unstable hydrophilic protein, NtDREB-1BL1 was located in the nucleus (Figure 2). ChIP-qPCR technology confirmed that the NtDREB-1BL1 could anchor the key segment of the *NtPSY* promoter (Figure 2), which was a major rate-limiting enzyme of carotenogenesis [46]. Overexpression of *NtDREB-1BL1* increased the expression of the *NtPSY* gene, thereby increasing the expression of genes in the carotenoid synthesis pathway. The contents of chlorophyll a, chlorophyll b, and carotenoid were increased in *NtDREB-1BL1 OE* transgenic plants, along with the decline in *NtDREB-1BL1 RNAi* transgenic tobacco (Figure 3 and Figure 4). This suggests that *NtDREB-1BL1* has a positive effect on carotenoids biosynthesis, indicating that the *NtDREB-1BL1* gene functions as an important transcriptional activator.

Drought stress inhibits plant root tip growth, leaf area, biomass accumulation, plant fresh weight and dry weight, causing serious losses to plant growth and development. When plants are subjected to drought and salt stresses, ROS are rapidly produced and accumulated in plant cells, resulting in severe oxidative damage. *DREB1A* in transgenic wheat participated in the process of plant drought resistance, which improved the drought resistance of transgenic plants [47]. *NtDREB-1BL1* was significantly and positively regulated by drought stress (Figure 5). This suggests that *NtDREB-1BL1* has role of defending against drought stress. Under drought stress, the ROS content of *NtDREB-1BL1 OE* transgenic tobacco was lower than that in the control, with the increase in ROS content in *NtDREB-1BL1 RNAi* transgenic tobacco (Figure 5). The lower levels of ROS may indicate that NtDREB-1BL1 conferred enhanced protection from oxidative damage and a better ROS scavenging ability in the transgenic plants. It suggested that NtDREB-1BL1 stimulated carotenoid accumulation in *N. tabacum*, which acted as essential antioxidants to scavenge ROS caused by drought stress. Our research provides a new idea and theoretical basis for the regulation of plant carotenoid biosynthesis. This study was able to provide new ideas and new understandings for the regulation of plant carotenoid biosynthesis.

## 5. Conclusions

In conclusion, we summarize NtDREB-1BL1 participates in regulating carotenoids biosynthesis by binding to the *NtPSY* promoter, which further enhances drought resistance of transgenic plants. Exploring the regulation of carotenoid biosynthesis by NtDREB-1BL1 will provide a new strategy of the genetic regulation of carotenoid content. Overall, our findings provide new insights into the characteristics and potential functions of NtDREB-1BL1 and offer a better understanding of their molecular basis in response to drought stress in *N. tabacum*.

## Data Availability

The data presented in the study are available in this manuscript.

## Supplementary Materials

The following supporting information can be downloaded at: https://www.mdpi.com/article/10.3390/genes13071134/s1, Table S1: Primer used in this study; Table S2: Information of DREB-1BL1 genes in *N. tabacum*, *N. tomentosiformis*, and *N. sylvestris*; Table S3: Distribution of Cis-element in *NtPSY* promoter.

## Abbreviations

PSY, phytoene synthase; DREB, dehydration-responsive element-binding protein; ChIP, chromatin immunoprecipitation; WT, wild-type; ROS, reactive oxygen species; MVA, the mevalonate pathway; MEP, the 2-C-methyl- D-erythritol-4-phosphate pathway; GGPP, geranylgeranyl diphosphate; PIF1, phytochrome-interacting factor 1; ERF, ethylene response element-binding protein; TUBB, *tubulin beta chain* genes; GA, gibberellin; DRE, dehydration responsive element; PDS, phytoene dehydrogenase; ZDS, zeta-carotene desaturase; CRTISO, carotenoid isomerase; LCYB, lycopene beta cyclase; LCYE, Lycopene epsilon cyclase; BCH, beta-carotene hydroxylase; VDE, violaxanthin de-epoxidase; NXS, neoxanthin synthase.
