# Supplementary material for: NtDREB-1BL1 Enhances Carotenoid Biosynthesis by Regulating Phytoene Synthase in Nicotiana tabacum
Source: Genes (Basel). 2022 Jun 24;13(7):1134. doi: 10.3390/genes13071134 (PMC9322988; doi:10.3390/genes13071134)
Supplement: Supplementary file 1 [file genes-13-01134-s001.zip › genes-1707535-supplementary.pdf]

**Table S1. Primer used in this study**

| Application | Primers | Sequence（5'-3'） |
|---|---|---|
| NtDREB-1BL1 isolation | NtDREB-1BL1-F | ATGGATATCTTTAGAAGCTATTATTCG |
| | NtDREB-1BL1-R | TTAGATAGAATAACTCCACAAAGGCATG |
| NtDREB-1BL1 OE | Sp1300-NtDREB-1BL1-F | TCTAGAATGGATATCTTTAGAAGCTATTATTC |
| | Sp1300-NtDREB-1BL1-R | GGTACCGATAGAATAACTCCACAAAGGC |
| NtDREB-1BL1 RNAi | RNAi-DREB-1BL1-F | GGGGACAAGTTTGTACAAAAAAGCAGGCTGCTATTATTCGGACCCACTTGC |
| | RNAi-DREB-1BL1-R | GGGGACCACTTTGTACAAGAAAGCTGGGTAGAACGGCCCCTTAATGCAAT |
| qPCR | NtDREB-1BL1-Q-F | ACCGACGCCAAGGATATTCAG |
| | NtDREB-1BL1-Q-R | ACAAAGGCATGTCAACATCAGC |
| | NtNtPSY1-F | TGTTGGAGAAGATGCCAGAAGAG |
| | NtNtPSY1-R | ATAAGCAATAGGTAAGGAAATTAGCTTC |
| | NtPDS-F | ATAAACCCTGACGAGCTTTC |
| | NtPDS-R | AATATGTTCAACAATCGGCAT |
| | NtZDS-F | GAGCATATGCGACAGGATCCCAC |
| | NtZDS-R | TGAAATAGGGGAGCTTGATTTCCGC |
| | NtCRTISO-F | CGTGTACACCGAGAATATGATG |
| | NtCRTISO-R | GTAGGCGAGAGTCAAGCACTC |
| | NtLCYB-F | GATGACAATACAACTAAAGATCTTGATAG |
| | NtLCYB-R | CATAAGCTACTTGATATCCAGGAT |
| | NtBCH-F | CTCAATTTTCATTTCAATCTCCTCTGTC |
| | NtBCH-R | ATGGCCGCCAGCAGAATTTC |
| | NtVDE-F | ATGATGCATGGGATGGATATG |
| | NtVDE-R | CGTTGGAGCTCTTTAAAACCTTC |
| | NtNXS-F | GCCGGGCTCTATTCGACGTGAT |
| | NtNXS-R | ACTCTACCATATGGTCTTCCCAAAT |
| | NtLCYE-F | CAGGAGTCTTTTTCGAGGAAACTTG |
| | NtLCYE-R | GTGTTCCAAGCTTGAGTTGAGAT |
| | 26S rRNA-F | GAAGAAGGTCCCAAGGGTTC |
| | 26S rRNA-R | TCTCCCTTTAACACCAACGG |
| | NtPSY-Frag1F | GTGGATGTGCGGGCATACA |
| | NtPSY-Frag1R | GCCAGTCGCTCACACCTCAT |
| | NtPSY-Frag2F | GCGGAGAGGTAGAGGGC |
| | NtPSY-Frag2R | CCTTCAACTTGTCGATTGG |
| | NtPSY-Frag3F | CCAATCGACAAGTTGAAGG |
| | NtPSY-Frag3R | ACGAGCTTAAGTTTTGTGAATTG |

| | | |
|---|---|---|
| ChIP-qPCR | NtPSY-Frag4F | CATTAGGGTATCGACACATTC |
| | NtPSY-Frag4R | TACATGTGATACCTCTAAGAGAAGC |
| | NtPSY-Frag5F | CCTCAATTCTATTTTGCTCCAT |
| | NtPSY-Frag5R | GCTTAGTGCACCGGACTACTC |
| | NtPSY-Frag6F | GTTATTTCCACGGCTCG |
| | NtPSY-Frag6R | CTTGTTGCAATTATGGTAGAGG |
| | NtPSY-Frag7F | CCTCTACCATAATTGCAACAAG |
| | NtPSY-Frag7R | CTAGTGCTTACTTTTCTAAATAGG |
| | NtPSY-Frag8F | TTAAACCAAAGCTTAGTGG |
| | NtPSY-Frag8R | AGGTGACTGAATAGTGTGAA |
| | NtPSY-Frag9F | TTCACACTATTCAGTCACCT |
| | NtPSY-Frag9R | AGTAACACAGTCAGTGCGG |
| | NtPSY-Frag10F | CCGCACTGACTGTGTTACT |
| | NtPSY-Frag10R | CTCTGTATAACAGCCTCCCTG |
| | NtTUBB1-F | AACTCTGATCTCCGCAAGCT |
| | NtTUBB1-R | TGGAACAGTCAAGGCTCGAT |
| | NtTUBB2-F | AGCTGCTAGTTCCAACGACT |
| | NtTUBB2-R | ATTCACGAAGGGTCCGACAA |

**Table S2.** Information of *DREB-1BL1* genes in *N. tabacum*, *N. tomentosiformis*, and *N. sylvestris*

| GENE ID | Gene Name | Mw (kDa) | PI | ORF (bp) | Amino acid | Coefficient of instability |
|---|---|---|---|---|---|---|
| XM_016632493.1 | *NtDREB-1BL1* | 24587.61 | 5.35 | 660 | 219 | 57.19 |
| *XM_009615919.3* | *NtomDREB1* | 24250.30 | 5.53 | 657 | 218 | 62.22 |
| *XM_009783485.1* | *N*sy*DREB1* | 24568.56 | 5.33 | 660 | 219 | 55.18 |

**Table S3.** Distribution of Cis-element in *NtPSY* promoter

| Region | Cis-element name | Function |
| --- | --- | --- |

| | | |
|---|---|---|
| 1st seg | TGA-element | auxin-responsive element |
| | TATA-box | core promoter element around -30 of transcription start |
| | CAAT-box | common cis-acting element in promoter and enhancer regions |
| | TCCC-motif | part of a light responsive element |
| 2nd seg | CAAT-box | common cis-acting element in promoter and enhancer regions |
| | G-box | cis-acting regulatory element involved in light responsiveness |
| | TGA-element | auxin-responsive element |
| | ABRE | cis-acting element involved in the abscisic acid responsiveness |
| 3rd seg | CAAT-box | common cis-acting element in promoter and enhancer regions |
| | TATA-box | core promoter element around -30 of transcription start |
| | TCT-motif | part of a light responsive element |
| | G-box | cis-acting regulatory element involved in light responsiveness |
| | ABRE | cis-acting element involved in the abscisic acid responsiveness |
| 4th seg | CAAT-box | common cis-acting element in promoter and enhancer regions |
| | TATA-box | core promoter element around -30 of transcription start |
| | G-box | cis-acting regulatory element involved in light responsiveness |
| | GA-motif | part of a light responsive element |
| | Box 5 | part of a conserved DNA module involved in light responsiveness |
| | ABRE | cis-acting element involved in the abscisic acid responsiveness |
| 5th seg | Box 6 | part of a conserved DNA module involved in light responsiveness |
| | TATA-box | core promoter element around -30 of transcription start |
| | GT1-motif | light responsive element |
| 6th seg | TATA-box | core promoter element around -30 of transcription start |
| | CAAT-box | common cis-acting element in promoter and enhancer regions |
| 7th seg | TATA-box | core promoter element around -30 of transcription start |
| | CAAT-box | common cis-acting element in promoter and enhancer regions |
| | GA-motif | part of a light responsive element |

| | | |
|---|---|---|
| 8th seg | A-box | cis-acting regulatory element |
| | CAAT-box | common cis-acting element in promoter and enhancer regions |
| | chs-CMA2a | part of a light responsive element |
| | TCT-motif | part of a light responsive element |
| | TATA-box | core promoter element around -30 of transcription start |
| 9th seg | CAAT-box | common cis-acting element in promoter and enhancer regions |
| | TATA-box | core promoter element around -30 of transcription start |
| | MBS | MYB binding site involved in drought-inducibility |
| 10th seg | ARE | cis-acting regulatory element essential for the anaerobic induction |
| | TATA-box | core promoter element around -30 of transcription start |
| | Box II | part of a light responsive element |
| | G-box | cis-acting regulatory element involved in light responsiveness |
| | GATA-motif | part of a light responsive element |
